# Intended and unintended impacts of the comprehensive reform of urban public hospitals: A mixed-method study in Hangzhou, China

**DOI:** 10.3389/fpubh.2022.979455

**Published:** 2022-10-10

**Authors:** Tao Zhang, Jing Liu, Beiyin Lu, Zhongheng Yan, Xiaojun Huang, Wei Lu

**Affiliations:** ^1^Department of Health Policy and Management, School of Public Health, Hangzhou Normal University, Hangzhou, China; ^2^Administrative Office, Yuebei People's Hospital, Shaoguan, China; ^3^Office of the Vice President, Central Hospital of Hainan Province Western, Haikou, China; ^4^Department of Health Management, School of Management, Hainan Medical University, Haikou, China; ^5^Hainan Women and Children's Health Care Centre, Haikou, China

**Keywords:** public hospital reform, health care expenditure, hospital revenue structure, panel-interrupted time-series, China

## Abstract

**Objectives:**

Public hospital reform is a key area in the Chinese healthcare system reform with the aim of controlling excessive growth of medical expenditures. This study aims to evaluate the impacts of two rounds of urban public hospital reforms respectively starting in 2018 and 2019.

**Method:**

A mixed-method method was conducted in Hangzhou. In the quantitative phase, monthly data covering 7 provincial, 12 municipal, and 35 district hospitals from March 2017 to June 2020 was analyzed using a panel-interrupted time-series. Thematic content analysis was conducted using qualitative data collected from 32 in-depth interviews.

**Results:**

Quantitative data showed a considerable reduction in the proportion of drug revenue (provincial hospitals: −4.937%; municipal hospitals: −2.765%; district hospitals: −2.189%) and an increase in the proportion of consumable (provincial hospitals: *β*_2_ = 2.025; municipal hospitals: *β*_3_ = 0.206) and examinations (provincial hospitals: *β*_2_ = 1.354, *β*_3_=0.159; municipal hospitals: *β*_2_ = 1.179) revenue after the first reform. In post-reform 2, The respective instant decrease and increase in the proportion of consumable (provincial hospitals: −2.395%; municipal hospitals: −0.898%) and medical services (provincial hospitals: 2.115%; municipal hospitals: −2.604%) revenue were observed. Additionally, quantitative and qualitative data indicated inpatient expenditures dropped considerably after the reform. However, insufficient compensation for medical services and increased financial pressure on hospitals were repeatedly mentioned as unintended consequences in qualitative interviews.

**Conclusions:**

Overall, the urban public hospital reforms in China created positive effects in adjusting hospital revenue structure and constraining soaring medical expenditures. Unintended consequences remind policymakers to establish rational and dynamic compensation mechanisms for public hospitals.

## Introduction

Over the past few decades, rapidly increasing health expenditures posed enormous challenges to China's healthcare system. Total health expenditures in China increased from 19980.4 billion yuan in 2010 to 65195.9 billion yuan in 2019, approximately equating to an average annual growth rate of 12.2%, which is higher than the annual gross domestic product (GDP) growth rate of 8.1% (1). Although contributors to soaring health expenditures are various (e.g., the aging population, increase in patients with chronic diseases, and advancements in medical technology), the effects of a compensation mechanism for public hospitals cannot be ignored (2, 3). Prior to 2009, the special policy of allowing public hospitals to gain additional profit from 15% drug mark-up was implemented to compensate for a long-time shortfall in government subsidy to public hospitals. Additionally, setting prices policy for healthcare services requiring large labor inputs are especially below costs (4). Under this distorted incentive system, hospitals usually awarded bonus physicians according to the number of drugs they prescribed, which resulted in the overuse of high-priced and unnecessary medicines. The data showed that revenue from drug sales in Chinese public hospitals accounted for 38.74% of total medical revenues in 2016 (5). As such, profits of public hospitals heavily relied on high-volume drug sales inevitably made a great contribution to the unreasonable growth in medical expenditures.

In order to reverse the compensation mechanism for public hospitals, the Chinese government started the public hospital reform and selected 311 county-level hospitals for pilot since 2012 (6). After the pilot projects, the government expanded the reform throughout the entire country in 2015, and further rolled it out to all urban public hospitals in 2017 (7). The reform consists of two core components: (1) the zero-mark-up drug (ZMD) policy removed the 15% profit margin for drug sales; (2) the pricing policy raised prices for medical services (8). In response to the central government's call for public hospital reforms, Zhejiang, an economically developed province located in southeast China, started to set up its policies based on the general guidance of the central government. In addition to ZMD and pricing policies, Zhejiang has also formulated several other policies in a stepwise manner since 2018. For example, adjusting the prices for high-tech diagnostic tests and increasing the outpatient consultation fee.

Overall, the continuously deepening reform of urban public hospitals in Zhejiang can be divided into two stages. In June 2018, Zhejiang launched the first round of reforms, including two core policies. The essential contents are as follows: (1) to minimize drug prices, drug procurement contracts attach procurement volumes to prices (namely “volume-price contract”) to attract suppliers to reduce pieces (9); (2) medical staff salaries were paid based on a provision of services (e.g., services quality and labor input). After this round of reform, however, unexpected consequences were reported in spite of some positive effects. For example, significant growth in examination and consumable expenditures (5, 10–12). Given these unwanted effects, the second reform was introduced in June 2019. Firstly, the types of medicines procured using volume-price contracts continued to expand, and a similar approach was employed in consumables procurement. Additionally, prices for 938 medical service items were further adjusted, including zero mark-up for consumables (ZMC), decreasing prices for examinations and laboratory tests, and increasing prices for services requiring professional skill and labor-intensive input (13).

Previous studies provided some evidence about the impacts of public hospital reform in China. For example, the ZMD policy was found to decrease inpatient medication spending significantly, but increase expenditures for diagnostic tests and medical consumables (11, 14). The unreasonable cost structures in hospitals were adjusted accordingly after pricing reform, while the large room for further optimization still existed (5, 15). However, we found there are several gaps to fill. Firstly, existing literature mainly focused on initial public hospital reforms, rarely involving new policies in the late reform period, such as volume-price contracts. Secondly, most of the studies used cross-sectional data, thus resulting in uncertain conclusions on the lasting-effect of reform (16, 17). Thirdly, differences in the response of hospitals at different levels to the reform are seldomly reported in the published literature.

To address these gaps, this study investigated how two rounds as mentioned earlier of public hospital reform in Zhejiang changed hospital expenditures and revenue structure, aiming to detect the intended and unintended impacts. Also, we examined whether the impacts varied depending on hospitals' level. The empirical evidence from this study made a contribution on policy implications for deepening reforms and also shed some light on other countries.

## Materials and methods

### Study design and setting

Taking Hangzhou, the capital city of Zhejiang province, as an example, this study employed the mixed method study to evaluate reform impacts. Hangzhou is located northwest of Zhejiang province and has a population of over 10 million residing on a land of 16,596 km^2^. In 2019, there were 72 urban public hospitals (14 municipal hospitals, 20 provincial hospitals), employing 78,163 health workers (18). These hospitals provided 3,77,10,151 outpatient and 10,82,550 inpatient visits, charging an average of 255.71 yuan for outpatients and 10718.54 yuan for inpatients (18).

### Quantitative phase

#### Data

The data for the quantitative study was collected from Zhejiang Public Hospital Reform Monitoring System, including hospital-level monthly data. This system covers all 72 urban public hospitals in Hangzhou. After excluding hospitals with missing and incorrect data, a full of 54 hospitals (7 provincial hospitals, 12 municipal hospitals and 35 district hospitals) were selected as the sample. According to the time cut point of the two rounds of reform, monthly data from March 2017 to June 2020 (pre-reform: March 2017–May 2018; post-reform 1: June 2018–June 2019; post-reform 2: July 2019–June 2020) was extracted to establish a balanced panel data for analysis.

### Outcome variables

Given data availability and the objectives of the public hospital reforms (19), indicators on the structure of hospital revenue and healthcare expenditure were designed and calculated. Revenue structure was measured by the proportion of four types of revenue in the total revenue: drug sales, consumable sales, examinations and medical services (20, 21). Average outpatient and inpatient expenditures per visit were used to assess the effect of reform on hospital expenditures (5).

### Statistical analysis

Panel-interrupted time-series (PITS) design for the three time periods was employed to evaluate changes in outcome variables due to the reform. As a quasi-experimental design, PITS predicts what outcomes would have been if there had been no reform based on levels and trends in pre-reform, and compares estimates with actual post-reform results (22, 23). The model is specified as follows:


(1) $$\begin{array}{l} Y_{t}=\beta_{0}+\beta_{1}*T_{t}+\beta_{2}*P_{1}+\beta_{3}*P_{1}*T_{t}+\beta_{4}*P_{2}+\beta_{5}*P_{2}*T_{t}+\varepsilon_{t} \end{array}$$

*Y*_*t*_ indicates the outcome variables in month t. *T*_*t*_ represents the time the study began. *P*_1_ and *P*_2_ indicate the two reforms, with a value of 0 denoting pre-reform and 1 denoting post-reform. *β*_0_ is the baseline level of outcome variables. *β*_1_ refers to the estimated value of the pre-intervention trend. *β*_2_ and *β*_4_ measure the level changes of outcome variables after the implementation of two reforms. *β*_3_ and *β*_5_ estimate the trend changes of outcome variables attributable to two reforms. *ε*_*t*_ refers to the error term.

According to the beginning time of the two rounds of public hospital reform, June 2018 and July 2019 were defined as two interruption points. Since the PITS dataset was generated by aggregating individual data by month, we employed the Newey-West method to handle autocorrelation and potential heteroskedasticity (22). Additionally, we also used Fourier terms (pairs of sine and cosine functions) to control for the seasonal effect and other long-term trends. To compare differences in the response of hospitals at different levels to the reform, provincial, the sample hospitals were divided into provincial, municipal and district-level hospitals to conduct PITS analysis separately. Stata V.14.0 was used for the statistical analysis.

### Qualitative phase

#### Sampling and interviews

Although PITS analysis provides precise estimates of policy impacts, there are some other policies implemented during the study period (March 2017–June 2020). A clear causal relationship between reform implementation and changes in outcomes variables might be interfered by these policies. As a result, a qualitative analysis was supplemented to verify the results of the empirical analysis. Moreover, a qualitative analysis was also used to explain the findings of quantitative analysis, and helped understand the internal mechanism of changes in outcomes after public hospital reform. We conducted the semi-structured interviews from September to October 2021 in Hangzhou. The study participants included health insurance administrators (*n* = 8), public hospital managers (*n* = 10), and physicians (*n* = 14) since these people have more knowledge about our topics. Purposive sampling approach was used to ensure a representative sample of men (*n* = 18) and women (*n* = 14), young (30–45 years, *n* = 13), middle (45–60 years, *n* = 8) and older (60+ years, *n* = 11) aged adults, and intermediate (*n* = 19) and senior title (*n* = 13) participants (24). After introducing the study aims and obtaining oral consent, each interviewee was asked to give their comments on the following three questions after being asked about demographic information: (1) “What positive effects do you think the policies in the public hospital reform created?”; (2) “What unexpected outcomes do you think public hospital reforms caused, and why?”; (3) “What barriers exist in the current reform of public hospital and how to remove them?”. A semi-structured interview guide was prepared and flexibly used to inform the interviews, while allowing space for new themes and views to emerge. However, respondents were reminded when their comments deviated from the theme of this study. One investigator was responsible for asking in the Chinese and the other was responsible for recording were assigned to conduct each interview. The face-to-face interviews were ~40 min in length and conducted in closed-door rooms. All the interviews were anonymized as soon as they were concluded. The key views of respondents were written down on the spot and verbatim by the interviewer. These comments were transcribed and synthesized by team members after the interviews.

### Content analysis

We used the thematic content analysis for respondents' opinions following principles of grounded theory performed on the NVivo 7.0 (25, 26). Firstly, two coders were assigned to read interview records and break the comments down into codes independently. Those comments outside the scope of this study were excluded in this stage. The consistency of the two sets of codes was tested using the kappa statistic (27). The coding agreement was 96.8%, while disagreements were resolved through further discussion. Then, we extracted those initial codes frequently mentioned (≥3 times) within the interviews and classified them into different categories depending on the similarity of meaning. Finally, after identifying the linkages between categories, dominant themes were generated accordingly with adequate discussion (28).

## Results

### Quantitative findings

Table 1 compared the monthly mean of outcome variables before and after the reforms by provincial, municipal and district hospitals. Overall, a certain reduction in the proportion of drug revenue and an increase in the proportion of medical services revenue were observed in these three categories of hospitals after the reforms. However, the proportion of examination revenue showed a growing trend after two rounds of reform. The proportion of consumable revenue presented a rise in post-reform 1 and a decline in post-reform 2. In terms of expenditures, per-inpatient expenditures dropped before and after reforms except for municipal hospitals, whereas outpatient expenditures increased slightly.

**Table 1:** Monthly mean of outcome variables before and after reforms by provincial, municipal, and district hospitals.

**Category**	**Indicator**	**Per-reform**	**Post-reform 1**	**Post-reform 2**
Provincial hospital	Drug revenue (%)	35.54	29.34	27.92
	Consumable revenue (%)	16.90	18.99	16.29
	Examinations revenue (%)	18.18	20.36	20.86
	Medical service revenue (%)	29.57	30.75	33.71
	Expenditures per outpatient visit (yuan)	299.61	333.63	361.05
	Expenditures per inpatient visit (yuan)	19617.21	18780.68	19525.05
Municipal hospital	Drug revenue (%)	33.71	30.10	28.77
	Consumable revenue (%)	16.24	17.46	12.94
	Examinations revenue (%)	23.44	25.19	25.59
	Medical service revenue (%)	25.78	29.35	34.33
	Expenditures per outpatient visit (yuan)	263.23	266.63	300.64
	Expenditures per inpatient visit (yuan)	15916.55	16262.04	16213.79
District hospital	Drug revenue (%)	32.32	28.56	31.14
	Consumable revenue (%)	12.68	12.98	9.61
	Examinations revenue (%)	26.81	28.33	28.49
	Medical service revenue (%)	27.67	30.02	31.73
	Expenditures per outpatient visit (yuan)	170.36	186.44	207.84
	Expenditures per inpatient visit (yuan)	9848.71	8921.08	9360.38

**Figure 1:**
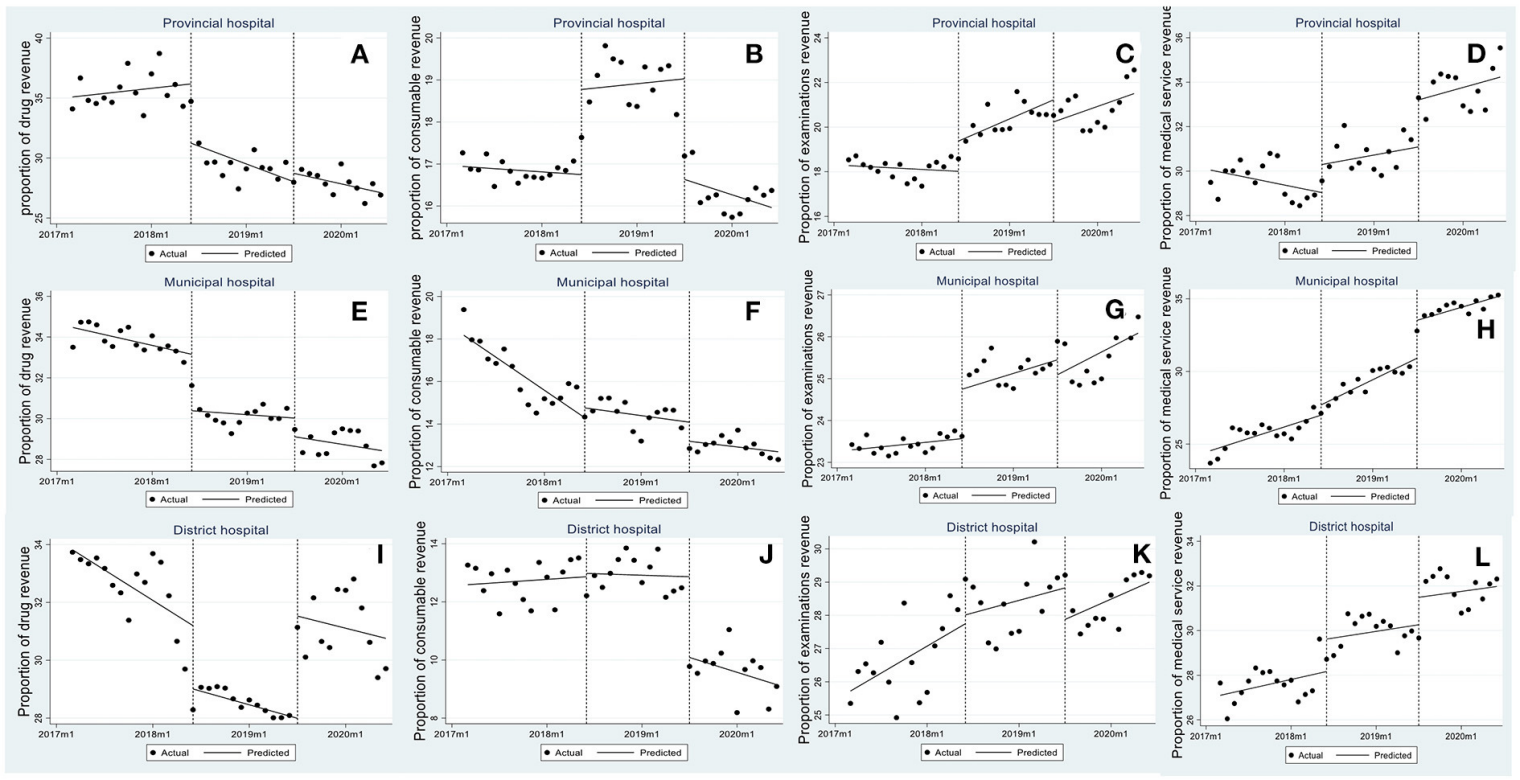
Trend in monthly revenue structure in provincial, municipal and district hospitals. **(A–D)** Proportion of drug revenue, consumables revenue, examinations revenue, and medical service revenue in provincial hospitals, **(E–H)** proportion of drug revenue, consumables revenue, examinations revenue, and medical service revenue in municipal hospitals, and **(I–L)** proportion of drug revenue, consumables revenue, examinations revenue, and medical service revenue in district hospitals.

Table 2 and Figure 1 showed PITS results for hospital revenue structure by different level hospitals. In terms of proportion of drug revenue, that in provincial hospitals (Figure 1A) dropped by 0.319% (*p* = 0.032) per month with immediate reduction of 4.937% (*p* = 0.005) in post-reform 1, that in municipal hospitals (Figure 1E) decreased by 2.765% (*p* < 0.001) and 0.916% (*p* = 0.024) in turn after two rounds of reforms, while that in district hospitals (Figure 1I) fell by 2.189% (*p* = 0.010) after first reform, but rose by 3.536% (*p* < 0.001) after second reform. The proportion of consumable (provincial hospitals: *β*_2_ = 2.025, *p* = 0.001; municipal hospitals: *β*_3_ = 0.206, *p* = 0.005) and examinations revenue (provincial hospitals: *β*_2_ = 1.354, *p* = 0.009; *β*_3_ = 0.159, *p* = 0.003; municipal hospitals: *β*_2_ = 1.179, *p* = 0.008) appeared to increase significantly after the first reform (Figures 1C,G), and then that of consumable revenue (provincial hospitals: *β*_4_ = −2.395, *p* < 0.001; municipal hospitals: *β*_4_ = −0.898, *p* = 0.024; district hospitals: *β*_4_ = −2.776, *p* < 0.001) experienced an immediate decline after the second reform (Figures 1B,F,J). In addition, the level change in the proportion of medical service revenue for provincial (Figure 1D) and municipal hospitals (Figure 1H) respectively increased by 2.115% (*p* = 0.001) and 2.604% (*p* < 0.001) after the second round of reform.

**Table 2:** Effects of reforms on hospitals' revenue structures based on PITS models.

**Category**	**Indicator**	**Post-reform 1**			**Post-reform 2**	
		**Baseline** **trend** **(*β_1_*)**	**Level** **change** **(*β_2_*)**	**Trend** **change** **(*β_3_*)**	**Level** **change** **(*β_4_*)**	**Trend** **change** **(*β_5_*)**
Provincial hospitals	Drug revenue	0.072	−4.937^*^	−0.319^*^	0.705	0.101
	Consumable revenue	−0.012	2.025^**^	0.032	−2.395^**^	−0.080
	Examinations revenue	−0.017	1.354^*^	0.159^*^	−0.988	−0.026
	Medical service revenue	−0.067	1.264	0.128	2.115^**^	0.032
Municipal hospitals	Drug revenue	−0.087^*^	−2.765^**^	0.059	−0.916^*^	−0.034
	Consumable revenue	−0.258^**^	0.463	0.206^*^	−0.898^*^	0.007
	Examinations revenue	0.017	1.179^*^	0.036	−0.356	0.036
	Medical service revenue	0.163^*^	0.705	0.084	2.604^**^	−0.097
District hospitals	Drug revenue	−0.175^*^	−2.189^*^	0.096	3.536^**^	0.008
	Consumable revenue	0.018	0.113	−0.027	−2.776^**^	−0.076
	Examinations revenue	0.146^*^	−0.088	−0.029	−0.668	0.006
	Medical service revenue	0.071	1.453	−0.021	1.228	−0.005

* p < 0.05;

** p < 0.001.

**Figure 2:**
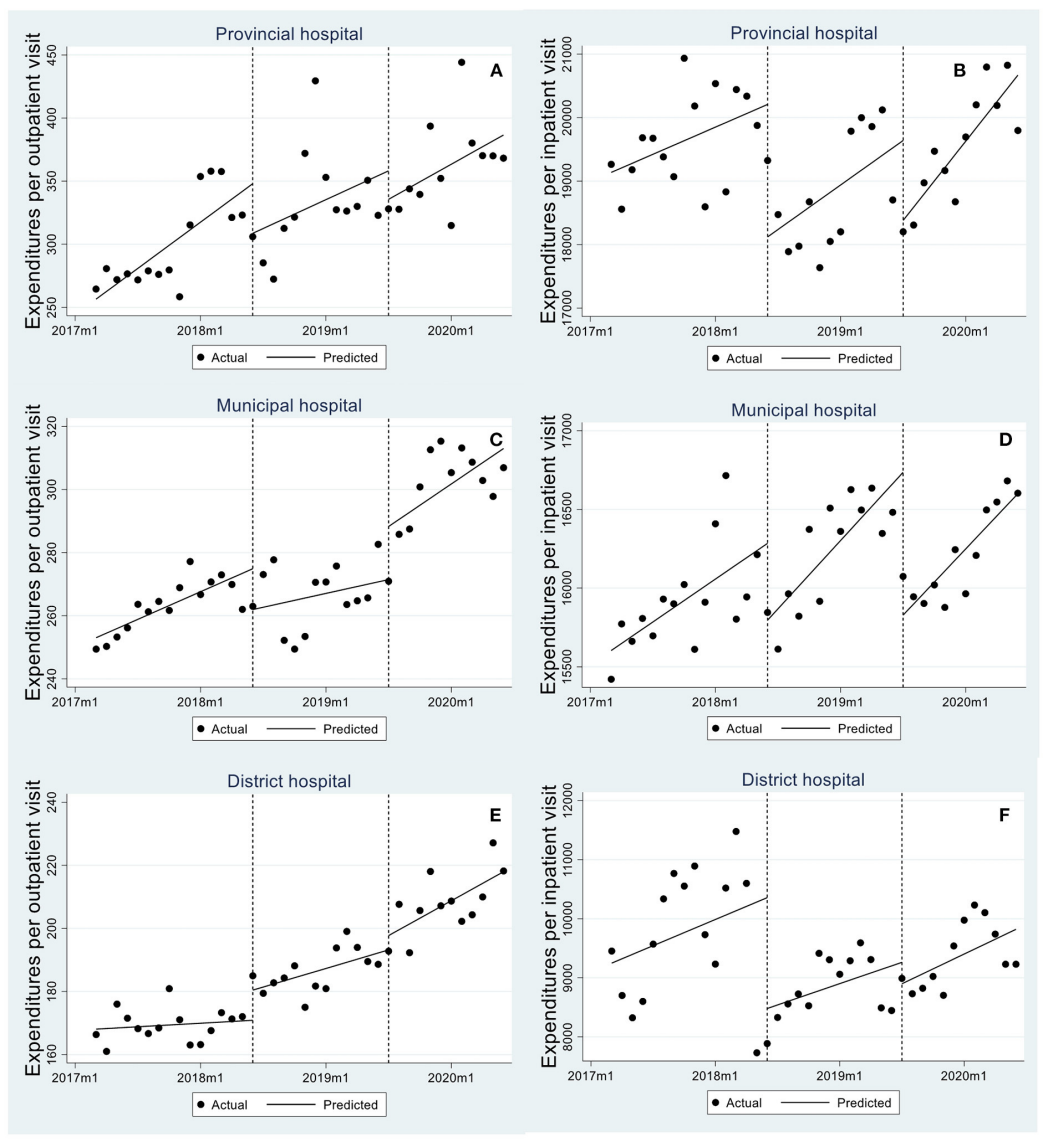
Trend in monthly expenditures per outpatient visit/hospitalization in provincial, municipal, and district hospitals. **(A,B)** are expenditures per outpatient visit/hospitalization in provincial hospitals, **(C,D)** are expenditures per outpatient visit/hospitalization in municipal hospitals, and **(E,F)** are expenditures per outpatient visit/hospitalization in district hospitals.

Table 3 and Figure 2 reported PITS results for outpatient and inpatient expenditures. After reform 1, inpatient expenditures per visit in provincial (Figure 2B), municipal (Figure 2D) and district hospitals (Figure 2F) respectively dropped by 2086.302 yuan (*p* = 0.001), 488.396 yuan (*p* = 0.018) and 1876.452 yuan (*p* = 0.025), whereas a slight growth in outpatient expenditures per visit (municipal hospital: *β*_2_ = 9.563, *p* = 0.004; district hospital: *β*_2_ = 9.559, *p* = 0.004) was observed (Figures 2C,E). After reform 2, average expenditures per hospitalization in provincial (Figure 2B) and municipal hospitals (Figure 2D) continued to reduce by 1259.293 yuan (*p* = 0.025) and 907.831 yuan (*p* < 0.001).

**Table 3:** Effects of reforms on outpatient and inpatient expenditures based on PITS models.

**Category**	**Indicator**	**Post-reform 1**			**Post-reform 2**	
		**Baseline** **trend** (***β_1_*)**	**Level** **change** **(*β_2_*)**	**Trend** **change** **(*β_3_*)**	**Level** **change** **(*β_4_*)**	**Trend** **change** **(*β_5_*)**
Provincial hospitals	Outpatient expenditures per visit	6.098^**^	−39.294	−2.294	−22.568	−0.828
	Inpatient expenditures per visit	71.407^*^	−2086.302^**^	45.410	−1259.293^*^	91.194
Municipal hospitals	Outpatient expenditures per visit	0.186	9.563^*^	0.793	8.056	−0.205
	Inpatient expenditures per visit	45.420^*^	−488.396^*^	26.871	−907.831^**^	−2.184
District hospitals	Outpatient expenditures per visit	0.187	9.559^*^	0.793	4.543	0.858
	Inpatient expenditures per visit	73.88	−1876.452^*^	−13.762	−362.862	23.662

* p < 0.05;

** p < 0.001.

### Qualitative findings

Although the quantitative analysis provided accurate estimates regarding the impacts of urban public hospital reforms, the qualitative interviews completed and enriched this picture. Table 4 reported qualitative findings. A total of six themes were identified and classified into two major domains: intended and unintended consequences.

**Table 4:** Content analysis results for effects of public hospital reform.

**Domains**	**Associated themes**	**Example of verbatim transcript**
Intended consequences	Reduced expenditure (26 interviewees)	“… After volume-price contracts, we found hospitalization expenditures for inpatients have dropped significantly, especially the expenditures of medicines…the economic burden is greatly reduced for those with chronic diseases who require long-term medication…” “… In the past, health insurance funds were under very pressure to balance their payments, but there was a slight surplus last year. And the growth rate of medical expenses is <10%, which is lower than the national average…”
	Shift in incentives for providers (18 interviewees)	“… The pricing reform aims to reduce the prices of medicines and consumables and raise the prices of medical services. Thus, it motived hospitals to reduce unnecessary drugs and consumables use and provide high-value services…. I think this incentive shift is beneficial for patients…”
	Improved services quality (13 interviewees)	“… It is difficult for us to gain profit from medicines and consumables after reform… We had to turn to high quality services to improve patient satisfaction and our competitiveness. For example, recently, Medical Service Quality Improvement Program was proposed in our hospital…”
Unintended consequences	Insufficient compensation for medical services (21 interviewees)	“… Although the reform emphasizes reducing the prices of medicines and consumables and increasing the prices of medical services, price adjustments are delayed, and compensation is inadequate. For example, the cost of medicines and consumables was reduced by 20% through volume-price contracts in March last year, but the price of medical services was only increased by 5% in June this year…”
	Increased financial pressure on hospitals (14 interviewees)	“… According to the hospital financial data in previous three years, the hospital's income has decreased significantly even a loss, due to removed profits from medicines and consumables. We faced great financial pressure though we have implemented many methods (e.g., clinical pathway) to reduce costs…”
	Unequal benefits among hospitals at different levels (11 interviewees)	“… Unlike tertiary hospitals, services in secondary hospitals are usually simple and low value. Thus, secondary hospitals cannot gain high profits when the reform increased prices of high technical and labor value services. It puts the secondary hospital in a very disadvantaged position…”

In terms of intended consequences, reduced expenditure was the most frequently mentioned. Respondents believed medical expenditures dropped greatly after reform because those medicines and consumables procurement using volume-price contracts were sold at a lower than past price. Additionally, policies to reduce the prices of medicines and consumables and increase the prices of services were considered to have changed previous incentives for hospitals to make profits from over-prescriptions. Some interviewees expressed public hospitals had to pay an emphasis on a high-quality service to gain profits in the future.

However, some unintended consequences were repeatedly mentioned during the interviews. For example, most respondents frequently complained that the slight increase in the price of medical services is not adequate to compensate for the hospital income loss resulting from the ZMD and ZMC policy. The public hospital reforms aim to shift the compensation mechanism in public hospitals from three financing sources (drugs/consumables, medical services and government subsidies) to two financing sources (medical services and government subsidy). Still, compensation from these two financing sources is insufficient to offset the operational cost, thereby resulting in the great financial pressure on public hospitals. Another place to be criticized is that price adjustment is not fair to secondary or district hospitals, because these hospitals are unable to provide high-tech medical services like tertiary hospitals. As such, the effects of reform often varied widely among hospitals at different levels.

## Discussion

Using PITS design supplementing with the qualitative interviews, this study assessed the impacts of urban public hospital reforms on provincial, municipal and district hospitals in Hangzhou, China. As expected, hospital revenue structure was generally optimized, and the medical expenditures were reduced after the reform of volume-price contracts in 2018 and the reform of price adjustment for medical service items in 2019. However, some unintended consequences are generated as well.

Consistent with previous findings (5, 20, 21), the PITS model showed a reduced proportion of drug revenue. Learning from the qualitative analysis, the implementation of volume-price contracts made a contribution to explain this finding. According to the theory of economies of scale, this new collective tendering and procurement arrangement for drugs generates dropped marginal costs with increased purchase volume (29, 30). In this case, manufacturers are required to sell contracted products to hospitals at a lower price. Accordingly, the price of drugs sold to patients in public hospitals dropped as well.

However, the proportion of consumables and examination revenue increased after the first reform. We assume that the public hospitals shifted profit sources in this period. On the one hand, the policy of volume-price contracts removed the profit from drug sales, but corresponding compensation through increasing prices for medical services is insufficient (31, 32). On the other hand, the policy of volume-price contracts has not been extended to the procurement of consumables in the first reform. As a result, providers were motivated to prescribe more examinations and use more consumables to offset the reductions in drug revenue (11). Encouragingly, the proportion of consumables showed an instant decline after the second reform. This change is attributable to subsequent reform for consumables procurement using volume-price contracts. These findings remind policymakers to implement measures to prevent providers' behaviors of seeking an inappropriate income source to compensate for revenue loss during the public hospital reform. For example, strict monitoring systems should be considered to put into practice.

Another notable finding from the quantitative analysis is that the increase in the proportion of medical services revenue is limited. Qualitative interviews also support this finding. Most respondents repeatedly mentioned that compensation for lost revenue by adjusting medical service prices is insufficient and delayed. A long time, the prices of medical service items in public hospitals were adjusted irregularly by the Chinese government (31, 32). However, the dynamic and scientific price adjustment mechanism was not established and put into practice, which resulted in the medical service pricing schedules were not updated in timely in most provinces following the pricing reform guidelines (21). The price of medical services is usually much lower than the labor value of medical staff. Policymakers should be informed that the range and extent of the price adjustment, especially for medical service items, should go further.

The data showed a non-significant change in the share of medical services revenue (Figures 1K,L), but an increased share of drug revenue in district-level hospitals after the second reform. Qualitative analysis helps to understand this finding. In the Chinese health system, district-level hospitals have poor resources and insufficient service capacity in comparison to that in provincial and municipal hospitals (33). These hospitals struggled to operate when pricing reform shifted the source of profits from sales of drugs and consumables to medical services. Under such circumstances, it was inferred that district-level hospitals were motivated to overuse medicines outside volume-prices contracts to compensate for the missing revenue. To ensure the fairness of reform benefits, pricing policy distinguishing hospital types should be taken into account.

Findings from both quantitative and qualitative analysis demonstrated the reforms achieved one of their set objectives of lowering medical expenditures. Two reasons are responsible for it. Firstly, the implementation of volume-price contracts made a remarkable contribution to the reduction in the cost of medicines and consumables (29, 30). Secondly, pricing reform reduced physicians' incentives of relying on medicines and consumables sales by shifting profit margins to medical services (34). This shift encourages providers to pay more attention to the improvement of service quality and efficiency (e.g., decreased length of stay, avoiding post-operative complications), which accordingly reached the effect of controlling total cost.

In addition to the above effects, we also found financial pressure on public hospitals increased substantially after the reform. Actually, many public hospitals' revenue reduced largely after the extra charge for drugs and consumables was banned, as well as the examination prices were downward lowered. Although the corresponding compensation for medical services by increasing their prices was allowed, it is far from enough to offset the operational cost (35). Simultaneously, the government subsidy has not significant increase after reform (36). Under such circumstances, it means that some public hospitals are struggling to operate, even in debt. This finding provides the policy implications that a dynamic compensation mechanism for public hospitals by raising medical service prices and increasing the government subsidy are recommended.

Several limitations in this study should be acknowledged. Firstly, there are some other policies implemented during the study period. These policies might interfere with the results of PITS models, further resulting in the fact that a clear causal relationship between reform implementation and changes in outcomes variables is unable to determine since the control group is not included in the analysis. Secondly, although all sample hospitals were continuously observed for 2 years after the reform, the COVID-19 pandemic in 2020 might change the disease composition in public hospitals, further interfering with our results. Thirdly, the restriction on data availability did not allow us to examine whether there is moral hazard and supplier-induced demand after reform, for example, patient selection and reduction in services. Fourthly, caution should be exercised in generalizing the empirical findings of this study, because we only used data from Hangzhou.

## Conclusion

The urban public hospital reforms in Hangzhou created encouraging effects in containing rising medical expenditures and optimizing hospitals' revenue structures by dropping the proportion of drug and consumable sales and raising the proportion of medical services. It indicates the reforms successfully achieved their aims as intended. However, some unintended consequences should be informed, for example, insufficient compensation for medical services and increased financial pressure on hospitals. The findings remind us that the establishment of a rational and dynamic compensation mechanism for Chinese public hospitals is extremely needed.

## Data availability statement

The raw data supporting the conclusions of this article will be made available by the authors, without undue reservation.

## Author contributions

TZ was responsible for the study design and implementation. BL collected and analyzed the data. WL reviewed and edited original draft. JL, ZY, and XH revised and critically commented the manuscript. All authors made significant contributions to this study. All authors read and approved the final manuscript.

## Funding

This study was supported by the Scientific Research Foundation for Scholars of HZNU (Grant number: 4265C50221204120), Research Fund of Hainan Medical University (Grant number: HYPY2020025), High-level Talents Project of Hainan Natural Science Foundation (Grant number: 821RC578), and Shaoguan City Social Development Technology Collaboration Innovation System Construction Project (Grant number: 220602104530735).

## Conflict of interest

The authors declare that the research was conducted in the absence of any commercial or financial relationships that could be construed as a potential conflict of interest.

## Publisher's note

All claims expressed in this article are solely those of the authors and do not necessarily represent those of their affiliated organizations, or those of the publisher, the editors and the reviewers. Any product that may be evaluated in this article, or claim that may be made by its manufacturer, is not guaranteed or endorsed by the publisher.
